# A novel signature of aging-related genes associated with lymphatic metastasis for survival prediction in patients with bladder cancer

**DOI:** 10.3389/fonc.2023.1140891

**Published:** 2023-06-27

**Authors:** Zhiguo Zhu, Xiaoli Li, Deqian Liu, Zhonghai Li

**Affiliations:** ^1^ Department of Urology, Affiliated Hospital of Jining Medical University, Jining Medical University, Jining, China; ^2^ Medical Research Center, Postdoctoral Mobile Station of Shandong University of Traditional Chinese Medicine, Jining, China; ^3^ The Seventh Affiliated Hospital (Shenzhen), Sun Yet-sen University, Shenzhen, China

**Keywords:** lymphatic metastasis, aging, extracellular matrix remodeling, prognostic signature, molecular subtypes, bladder cancer

## Abstract

**Background:**

The predominant and most prevalent form of metastatic bladder cancer (BCa) is lymphatic metastasis, which is associated with a highly dismal prognosis for patients. Aging-related genes (ARGs) are believed to contribute significantly to tumor development. However, the effect of ARGs on lymphatic metastasis of BCa is unclear. This research sought to establish a prognosis model based on ARGs associated with lymphatic metastasis in BCa.

**Methods:**

We downloaded BCa data from the TCGA and GEO databases and ARGs from the Aging Atlas database. The least absolute shrinkage and selection operator (LASSO) approach was applied to obtain the characteristic ARGs of risk signature in the TCGA cohort. Verification was done using the GSE13507 dataset. The R package ‘ConsensusClusterPlus’ was employed to identify the molecular subtypes based on the characteristic ARGs. Protein-Protein interaction network, MCODE analysis, enrichment analysis (KEGG, GO, GSEA), and immune infiltration analysis were performed to investigate underlying mechanisms. EdU, migration and invasion assays, wound healing assays, immunofluorescence staining, and quantitative polymerase chain reaction were conducted to evaluate the impact of ELN on the proliferative, migratory, and invasive capacities of BCa cells.

**Results:**

We identified 20 differently expressed ARGs. A four ARGs risk signature (*EFEMP1*, *UCHL1*, *TP63*, *ELN*) was constructed in the TCGA cohort. The high-risk group (category) recorded a reduced overall survival (OS) rate relative to the low-risk category (hazard ratio, 2.15; P <0.001). The risk score could predict lymphatic metastasis in TCGA cohort (AUC=0.67). The GSE13507 dataset was employed to verify the validity of this risk score. Based on the four ARGs, two distinct aging profiles (Cluster 1 and Cluster 2) were discovered utilizing the ConsensusClusterPlus, and Cluster 2 possessed a favorable OS in contrast with Cluster 1 (hazard ratio, 0.69; P =0.02). Classical tumor signaling pathways, ECM-associated signaling pathways, and immune-related signaling pathways participate in BCa progression. ELN recombinant protein affected the expression of collagen and increased migration and invasiveness in BCa cells.

**Conclusion:**

We constructed a four-ARG risk signature and identified two aging molecular subtypes. This signature could serve as an effective survival predictor for patients with BCa.

## Introduction

Bladder cancer (BCa) is among the most prevalent types of cancer in the urinary system. In 2022, it was projected that there would be 81,180 newly diagnosed cases of BCa in the United States, with 17,100 deaths attributable to this disease (1). Two subtypes of BCa have been established, hallmarked by the extent to which they have invaded muscles: non-muscle-invasive bladder cancer (NMIBC) and muscle-invasive bladder cancer (MIBC). Generally, transurethral excision of the bladder tumor is a viable treatment option for 70% of individuals diagnosed with NMIBC (1). Prognosis in individuals with MIBC is most strongly influenced by lymphatic metastasis, the most predominant type of BCa metastasis (2, 3). The survival rate of MIBC individuals is considerably reduced (15-31% vs. >60%) if lymphatic metastasis is present (1, 4). Consequently, it is a good option to build a risk signature for survival predictor based on lymphatic metastasis samples.

Predictive and prognostic models can facilitate the establishment of more personalized form of treatment. Increased genomic knowledge is transforming BCa diagnosis and treatment. Apoptosis, autophagy, immune regulation, DNA methylation, and alterations of chromatin conformation are involved in the initiation and progression of bladder cancer (5). Researchers have established some risk models based on these biological processes to predict recurrence, metastasis, drug resistance and survival of patients with cancer. Such as, Chen et al. (6) constructed an immune checkpoint signature to predict patient prognosis and immunotherapy responsiveness. In addition, genitourinary tumors are especially suitable for urine assay. Chen et al. (7) developed an efficient urine DNA methylation assay method which enables early detection and recurrence monitoring for BCa.

Aging is the phenomenon in which cells lose their ability to proliferate and enter a state of growth arrest, first reported by Hayflick in 1961 (8). Cell senescence is widespread in the body and runs through the whole life stages. It performs a fundamental physiological function in embryonic development and tissue repair. Aging is characterized by a permanent cessation of proliferation. Therefore, it is generally regarded as a tumor suppressive process, and a key effector mechanism of many types of anticancer therapies (9–11). However, with the deepening of research, researchers found that senescence-associated reprogramming promotes cancer stemness (12). Senescent cells can enhance tumor progression by modulating the tumor microenvironment through a mechanism known as the senescence-associated secretory phenotype (SASP). Therefore, we sought to establish a prognosis model based on ARGs associated with lymphatic metastasis in BCa.

Herein, we successfully constructed a four-ARG risk signature and identified two aging molecular subtypes with significant prognostic differences. ELN recombinant protein affected the expression of collagen and increased the migratory and invasive capacities of BCa cells.

## Materials and methods

### Data selection

The TCGA dataset (n=426) was extracted from the UCSC Xena database (http://xena.ucsc.edu/), and the GSE13507 dataset (n=165) was derived from the GEO database (https://www.ncbi.nlm.nih.gov/geo/). In the Aging Atlas database (13), 502 ARGs were obtained. TCGA cohort included 365 patients with definite regional lymphatic staging data (N0, n=236; N1-3, n=129). We analyzed gene expression using these data and developed an innovative prognostic signature.

### Discovery of differentially expressed ARGs

Differentially expressed genes (DEGs) across lymphatic metastasis negative and positive samples were extracted using the “LIMMA” program, with the adjusted P-value < 0.05 and fold change (FC) > 1.5 or FC < 0.67 as the thresholds. We visualized DEG results using volcano plots and heat maps. DEGs and ARGs were intersected to identify differentially expressed ARGs.

### ARGs risk signature construction

Through univariate Cox regression analysis, we found the DEGs linked to aging that were correlated with OS. The R package ‘glmnet’ (14) and least absolute shrinkage and selection operator (LASSO) technique were applied to obtain the characteristic genes of risk signature. The calculation formula of the model is as indicated below: risk score = sum (each gene’s expression × corresponding coefficient). We determined which patients were at high risk and those at low risk as per their risk scores. Differences (variations) in OS between the two risk categories were analyzed using the Kaplan-Meier (KM) technique. Risk signature stability was verified at 1-, 3-, and 5-year periods using time-dependent receiver operating characteristic (ROC) curves.

### Identification of aging molecular subtypes

The ‘ConsensusClusterPlus’ package (15) was employed to discover the aging molecular subtypes. Variations in OS among various aging molecular subtypes were examined via the KM analysis. The accuracy of risk scores and ARGs in classifying aging subgroups was assessed using the ROC curve.

### Pathway and process enrichment analysis and PPI network analysis

Enrichment analysis was performed with the help of Metascape (http://metascape.org) (16). A protein-to-protein interaction (PPI) network representing the interplay between DEGs was generated with the assistance of the STRING database and the Cytoscape program. To filter the PPI network’s modules, the Molecular Complex Detection (MCODE) plug-in of Cytoscape was utilized.

### Gene set enrichment analysis

GSEA (17) was conducted to investigate the variations in biological processes between diverse subgroups. To evaluate the pathways and relevant molecular processes, the gene sets known as ‘c2.cp.kegg.v7.4.symbols.gmt’ were extracted from the Molecular Signatures Database. The significance criterion was established at P < 0.05.

### Immune infiltrations analysis

CIBERSORTx (18) is an analytical tool, capable of conducting linear support vector regression to estimate immune cell infiltration, using gene expression data. We calculated 22 distinct immune cells in patients using CIBERSORTx and analyzed the correlation and difference between the proportion of various immune cells, the abundance of each type of immune cell, and gene expression. A P < 0.05 denoted the significance criterion.

### Correlation of genes with signal pathways

Four signal pathways, epithelial-mesenchymal transition (EMT), ECM, degradation of ECM, and collagen formation, were selected to analyze their association with characteristic ARGs of risk signature. RNA-sequencing expression (level 3) profiles and relevant clinical data for BCa were derived from the TCGA dataset (https://portal.gdc.com). After collecting the genes that were found in the respective pathways, the ‘GSVA’ R package was used to examine the scores of the pathways, and lastly, the association of gene expression with pathway scores was investigated using the Spearman method.

### Cell culture

Human BCa cell line SW780 was presented by Yong Xia from Jining Medical University. Cells were grown in RPMI-1640 (Gibco, Carlsbad, CA, USA) comprising 10% FBS (ExCell Bio, Shanghai, China), 1% streptomycin, and penicillin (Gibco, Carlsbad, CA, USA) at 37 °C and 5% CO2. Coating cell culture plates with human recombinant ELN (10μg/ml, E6902, Sigma, St. Louis, USA) that had been solubilized in sterile PBS was done before overnight incubation. Plates serving as controls were only coated with PBS. The cells were used for subsequent experiments after they had been cultured in ELN-coated cell culture plates for 48hrs.

### Cell proliferation assay

By following the directions stipulated by the manufacturer of the EdU staining kit (Beyotime Biotechnology, Shanghai, China), we conducted the cell proliferation experiment. EdU-positive rate = EdU-positive cell count/total cell count.

### Migration and invasion assays

Evaluations of the capacity of PCa cells to migrate in two dimensions were carried out using wound healing tests. The Transwell chamber (Corning, NY, USA) was employed to evaluate cell migration and invasiveness at a 3D level. Each experiment was conducted in compliance with the guidelines stipulated by the manufacturer.

### Quantitative real-time PCR

As recommended by the manufacturer of the TRIzol Reagent (Invitrogen; Thermo Fisher Scientific, Inc., Carlsbad, CA, USA), we extracted total RNA from the cells. Subsequently, the PrimeScript RT reagent Kit with gDNA Eraser (Takara, Beijing, China) was used in the synthesis of the cDNA derived from the mRNA, and the SYBR green Premix Ex Taq II (Takara) was utilized to conduct qPCR on the Applied Bio-systems 7500 Fast Real-Time RCR System (Applied Biosystems, Foster City, CA, USA). The ^△△^Ct method was adopted to quantify the relative mRNA level. Primer information used in the study can be found in **Supplementary Table S1**.

### Immunofluorescence staining

IF staining was carried out as previously described (19) using antibodies against COL1A1 (Proteintech, Wuhan, Hubei, China, # 67288-1-Ig) at dilution of 1:200. Using Image J, we computed the mean fluorescent intensity.

### Statistical analysis

SPSS v 20.0, Graphad prism v 8.0, as well as R software v 4.0.3 and corresponding packages, were used to conduct all the analyses of statistical data. We established the significance criterion as *P* < 0.05.

## Results

### Dysregulation of ARGs in lymphatic metastatic of BCa

**Figure 1:**
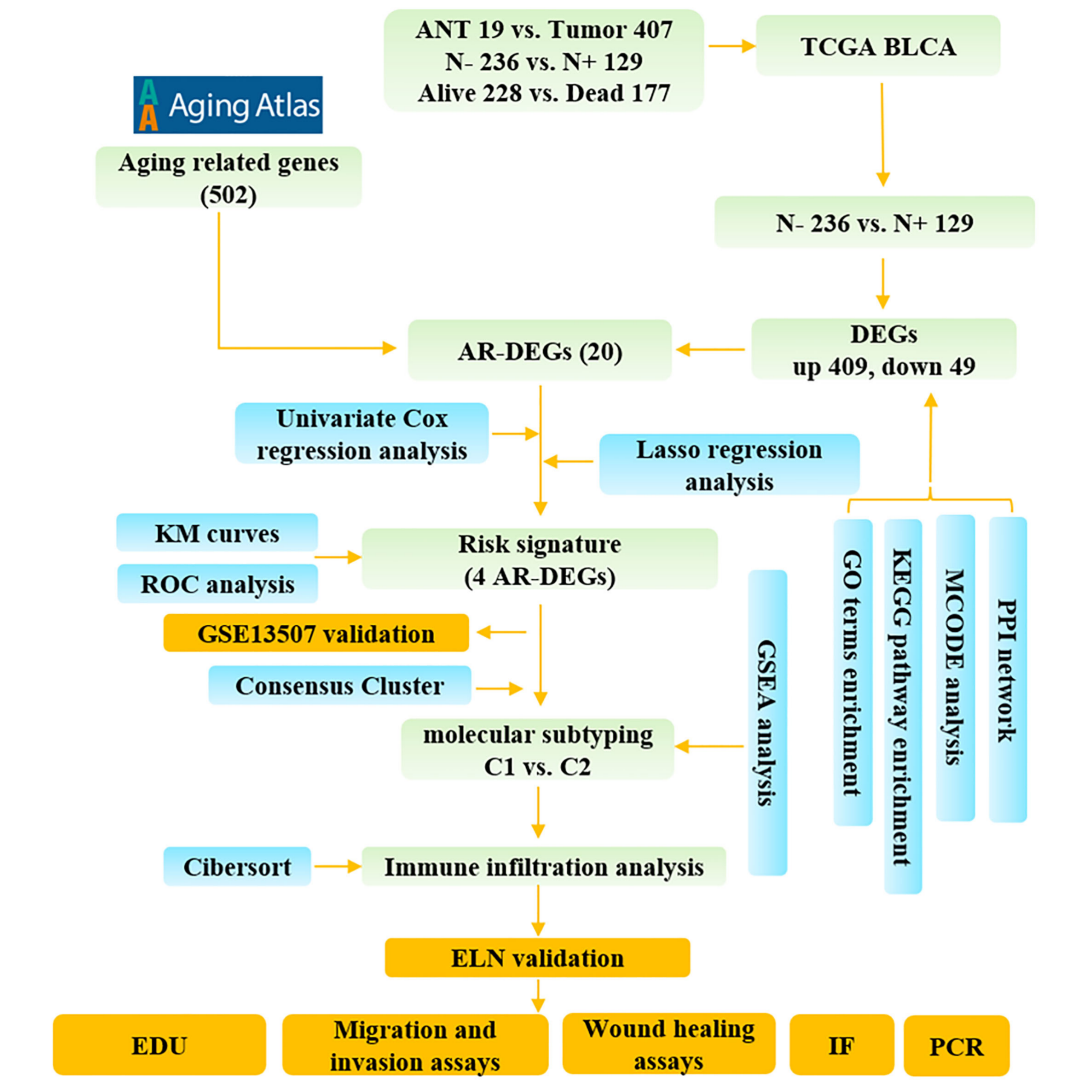
The study flow diagram. ANT, adjacent normal tissue; N-, lymphatic metastasis negative; N+, lymphatic metastasis positive; DEGs, differentially expressed genes; ARGs, aging-related genes; AR-DEGs, aging-related DEGs; KM, Kaplan–Meier; ROC, receiver operating characteristic; GO, Gene Ontology; KEGG, Kyoto Encyclopedia of Genes and Genome; PPI, protein-protein interaction; GSEA, Gene set enrichment analysis; IF, immunofluorescence staining.

**Figure 2:**
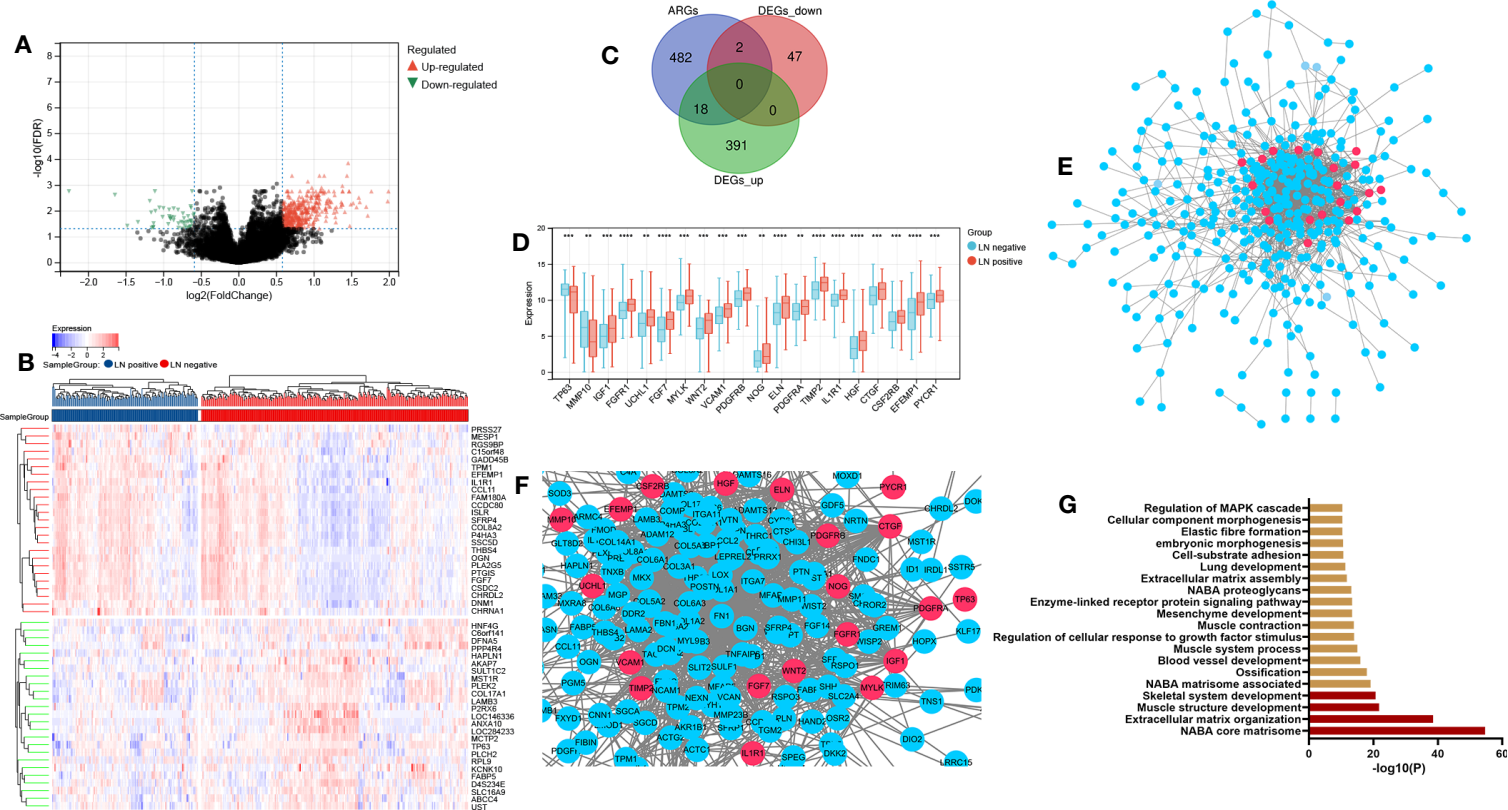
Dysregulation of ARGs in lymphatic metastatic of BCa. **(A)** P-adjust and fold change values were used in the development of the volcano plot.; up-and-down-regulated genes are displayed correspondingly by red and blue. **(B)** The top 50 DEGs are displayed on a heatmap. **(C)** Aging genes versus DEGs Venn diagram. **(D)** The expression histogram of 20 AR-DEGs. **(E, F)** DGEs PPI network: red nodes indicate AR-DEGs. **(G)** Functional enrichment analysis. N-, lymphatic metastasis negative; N+, lymphatic metastasis positive; DEGs, differentially expressed genes; ARGs, aging-related genes; AR-DEGs, aging-related DEGs; PPI, protein-protein interaction. Data are expressed as means +/- standard deviation. *P < 0.05; **P < 0.01; ***P < 0.001; ****P < 0.0001.

**Figure 3:**
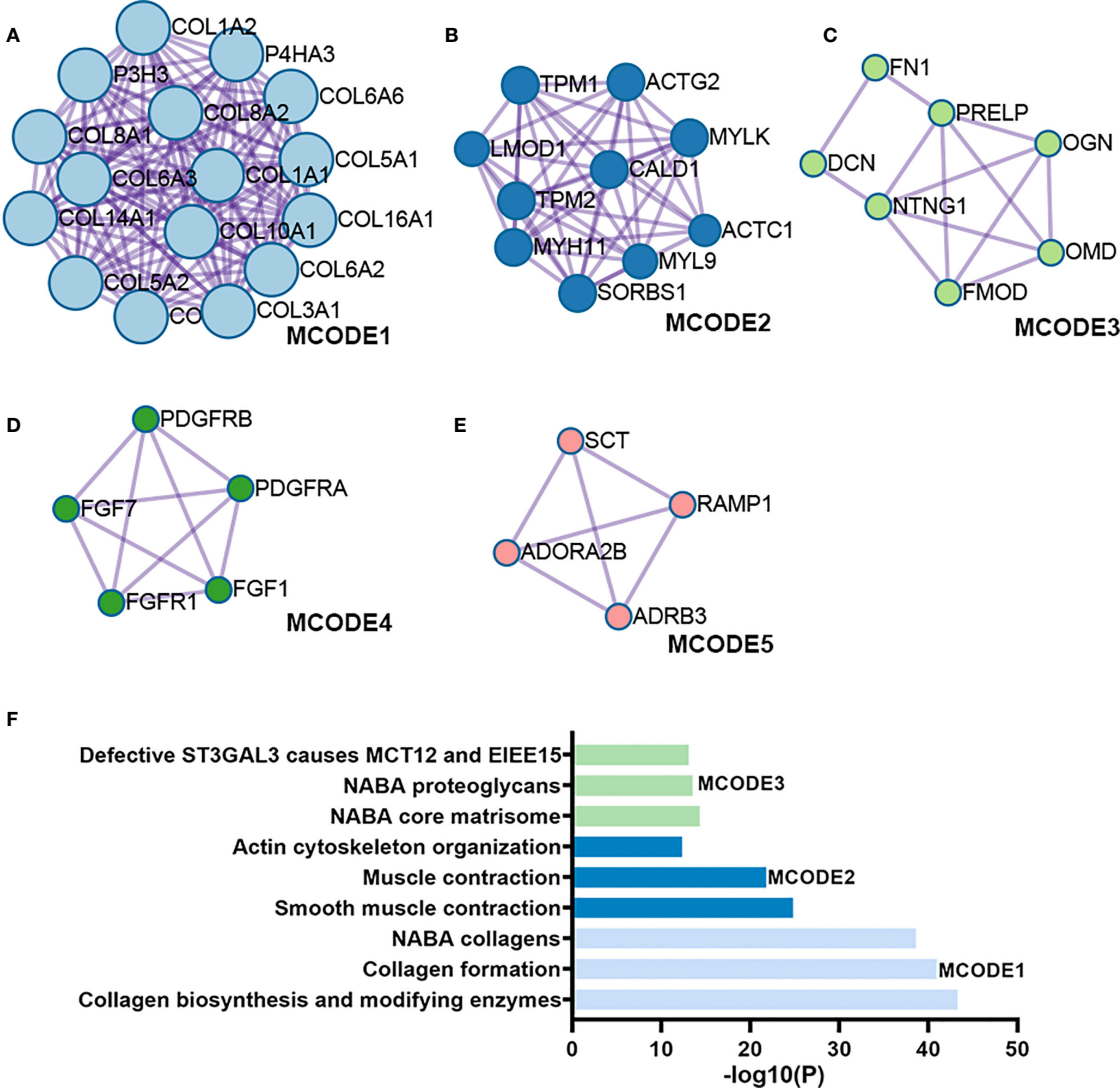
Module analysis of DEGs. **(A-E)** Five modules screened from the PPI network. **(F)** Functional enrichment analysis.

**Figure 4:**
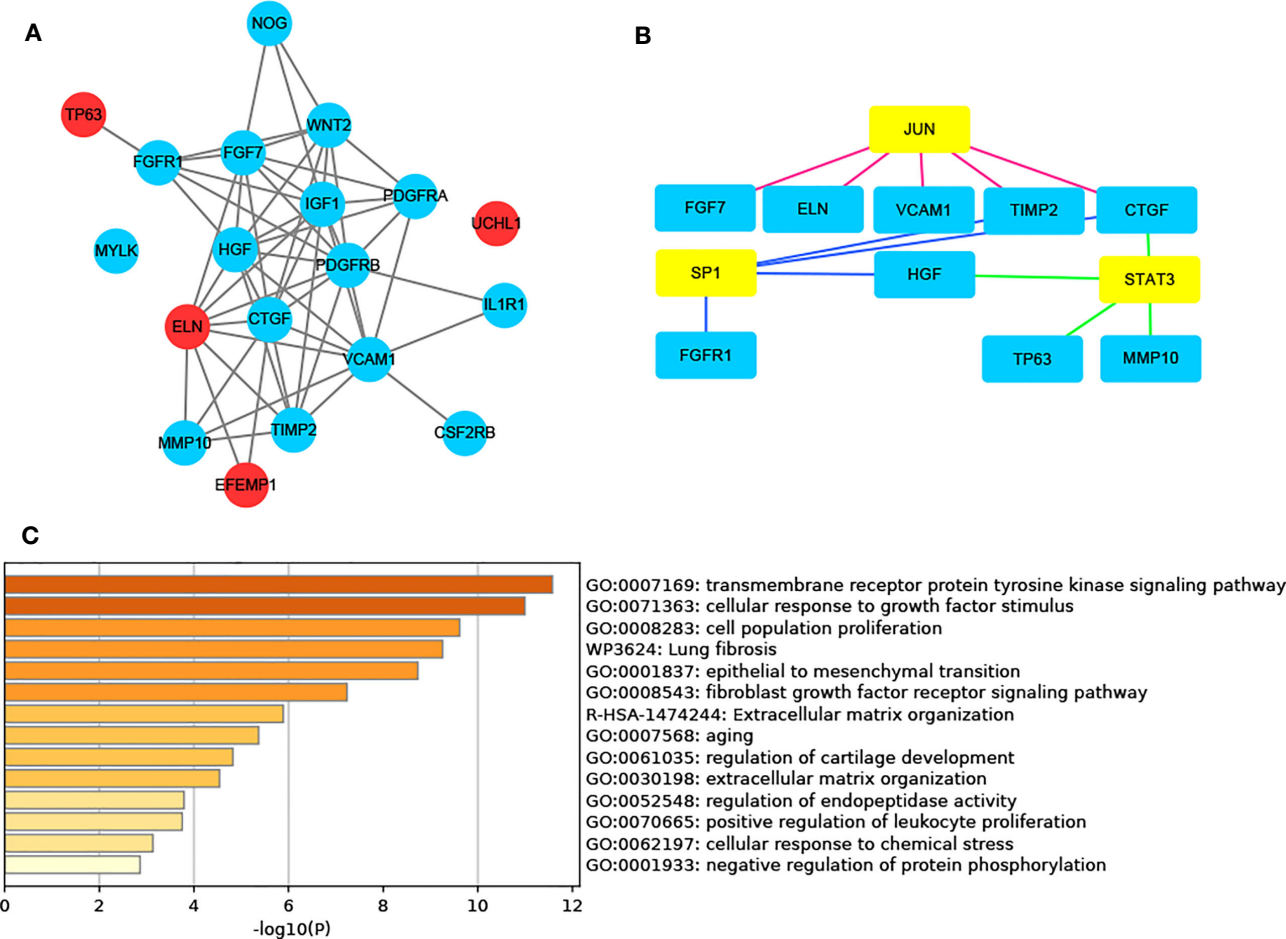
AR-DEG-related functional analysis and PPI network. **(A)** AR-DGEs PPI network: red nodes indicate four signature ARGs. **(B)** Transcription factor regulatory network: yellow nodes indicate transcription factor. **(C)** Functional enrichment analysis. ARGs, aging-related genes; AR-DEGs, aging-related DEGs; PPI, protein-protein interaction.

**Figure 1** illustrates the process for collecting and analyzing data. In the beginning, the RNA sequencing data of BCa was downloaded from TCGA along with matching clinical information. We screened out 458 DEGs (409 up-regulated and 49 down-regulated) in TCGA cohort patients with lymphatic metastases and those without (**Figures 2A–C**). When DEGs and ARGs overlapped, 20 aging-related DEGs (AR-DEGs) were found, 18 of which were upregulated and two downregulated (**Figure 2C**). The expression patterns of these AR-DEGs are displayed as histograms in **Figure 2D**. To further understand the links among DEGs, we searched the STRING database to formulate a PPI network of DEGs and evaluated their relationships using Cytoscape software. The PPI network of DEGs shows that the distribution of 20 AR-DEGs was scattered (**Figure 2E**). **Figure 2F** is a partially enlarged display of the PPI network. Then, we used Metascape to perform annotation and enrichment analysis of DEGs, and **Figure 2G** demonstrated the top 20 clusters as a clustered heatmap. Furthermore, enrichment of the DEGs was revealed in NABA signaling pathways, ECM organization, and organismal development. MCODE plugin was also utilized to find highly connected nodes in the network. **Figures 3A–E** display the compiled MCODE networks that were discovered for various gene sets. We performed enrichment analysis on the top 3 MCODEs (**Figure 3F**). Mcode1 was mainly composed of collagen genes, and enriched in collagen formation. Mcode2 was enriched in organismal development, and Mcode3 was enriched in NABA signaling pathways. **Figure 4A** displays the PPI network of AR-DEGs. Through TRRUST, we got 3 transcription factors (*JUN*, *SP1*, *STAT3*) that regulate AR-DEGs (**Figure 4B**). These AR-DEGs were primarily enriched in ECM organization and aging signaling pathways (**Figure 4C**).

### Construction and verification of the ARGs risk signature

**Figure 5:**
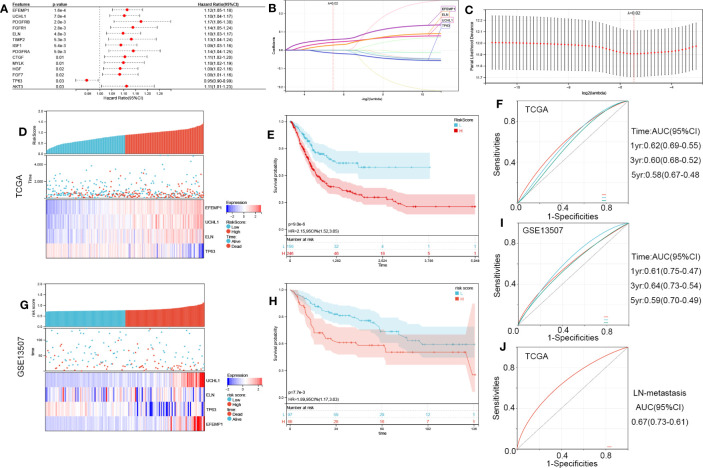
Construction of the four-ARG risk signature. **(A)** Identification of 14 aging-related DEGs linked to the OS of BCa patients through univariate Cox regression analysis in TCGA. **(B, C)** Screening out four characteristic genes out of 14 ARGs using the LASSO algorithm. **(D)** The Riskscore, survival time, and survival status of BCa in TCGA. **(E)** Analysis of the two risk categories using Kaplan-Meier survival analyses. The TCGA cohort was classified into high- and low-risk categories as per the median risk score. **(F)** Time-dependent ROC curves verified the prognosis-predictive efficacy of the risk signature at 1, 3, and 5 years. **(G-I)** the validation in the GSE13507 cohort. **(J)** ROC curves verified the LN-metastasis predictive efficacy of the risk signature. ROC, receiver operating characteristic; LASSO, least absolute shrinkage, and selection operator; ARGs, aging-related genes.

We identified 14 AR-DEGs correlated with the OS of patients via univariate Cox regression (**Figure 5A**). Only *TP63* served as a protective factor. To prevent overfitting of the model, the LASSO technique was employed to choose four signature genes from a total of 14 AR-DEGs (**Figures 5B, C**).

**RiskScore**=0.0574554179357342* ***EFEMP1*** + 0.0426771361572733* ***UCHL1*** + 0.0273704531574397* ***ELN***-0.0161007481578848* ***TP63***

We classified BCa samples into high-risk and low-risk categories as per their median risk score (**Figure 5D**). KM survival curves revealed that the high-risk patients possessed a poor OS (HR, 2.15; 95% CI, 1.52-3.05; P<0.001; **Figure 5E**). The effectiveness of the risk signature as a predictive tool is illustrated in **Figure 5F**. GSE13507 was employed to validate the validity of this risk score (**Figures 5G–I**) and showed good robustness. ROC curves were used to verify the LN-metastasis predictive efficacy of the risk signature (**Figure 5J**, AUC=0.67).

### Identification of aging molecular subtypes

**Figure 6:**
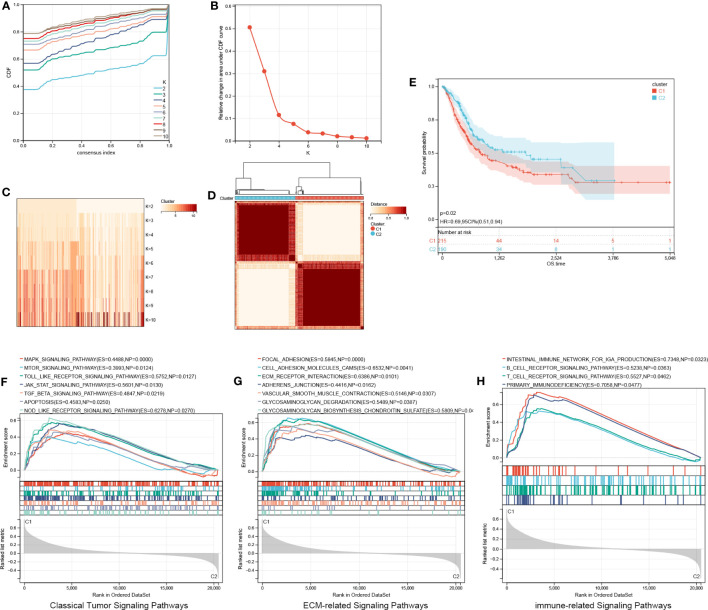
Identification of aging molecular subtypes. **(A–D)** Two aging molecular subtypes obtained using ConsensusClusterPlus. **(E)** The two aging molecular subtypes were analyzed via a Kaplan-Meier survival analysis. **(F–H)** GSEA on all genes between two aging patterns. GSEA, Gene set enrichment analysis.

Based on four signature ARGs, two aging molecular subtypes (cluster 1 and cluster 2) were discovered utilizing the R package ‘ConsensusClusterPlus’ (**Figures 6A–D**). In total, 215 samples were found in cluster 1, whereas 190 were found in cluster 2. KM survival curves revealed that cluster 2 possessed a favorable OS (HR, 0.69; 95% CI, 0.51-0.94; P =0.02; **Figure 6E**). We performed GSEA on all genes between the two aging categories. The results revealed that classical tumor signaling pathways, ECM-related signaling pathways, and immune-associated signaling pathways were differentially expressed in the molecular BCa subtypes (**Figures 6F–H**).

**Figure 7:**
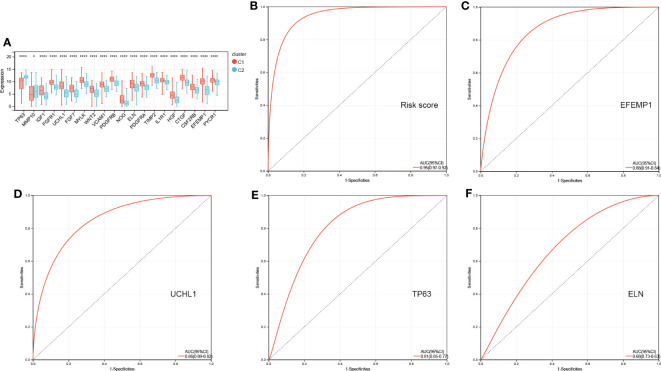
ROC curves of risk score **(B)** and four characterized genes **(C-F)** independently distinguished between cluster 1 and cluster 2. **(A)** The expression histogram of 20 AR-DEGs in two aging molecular subtypes. ROC, receiver operating characteristic; AR-DEGs, aging-related DEGs. Data are expressed as means +/- SD. *P < 0.05; ****P < 0.0001.

**Figure 7A** shows 20 AR-DEG expression levels in the two subtypes. Cluster 2 showed considerably elevated TP63 and MMP10 expression levels relative to cluster 1, whereas other genes showed remarkably decreased expression levels in cluster 1 compared to cluster 2. Moreover, the ROC curves were generated to assess the prognostic value of the risk score and each of the four ARGs, and the findings showed that all of them, especially the risk score, had good classification efficacy for the two aging molecular subtypes (**Figures 7B–F**).

### Differences in immune characteristics

**Figure 8:**
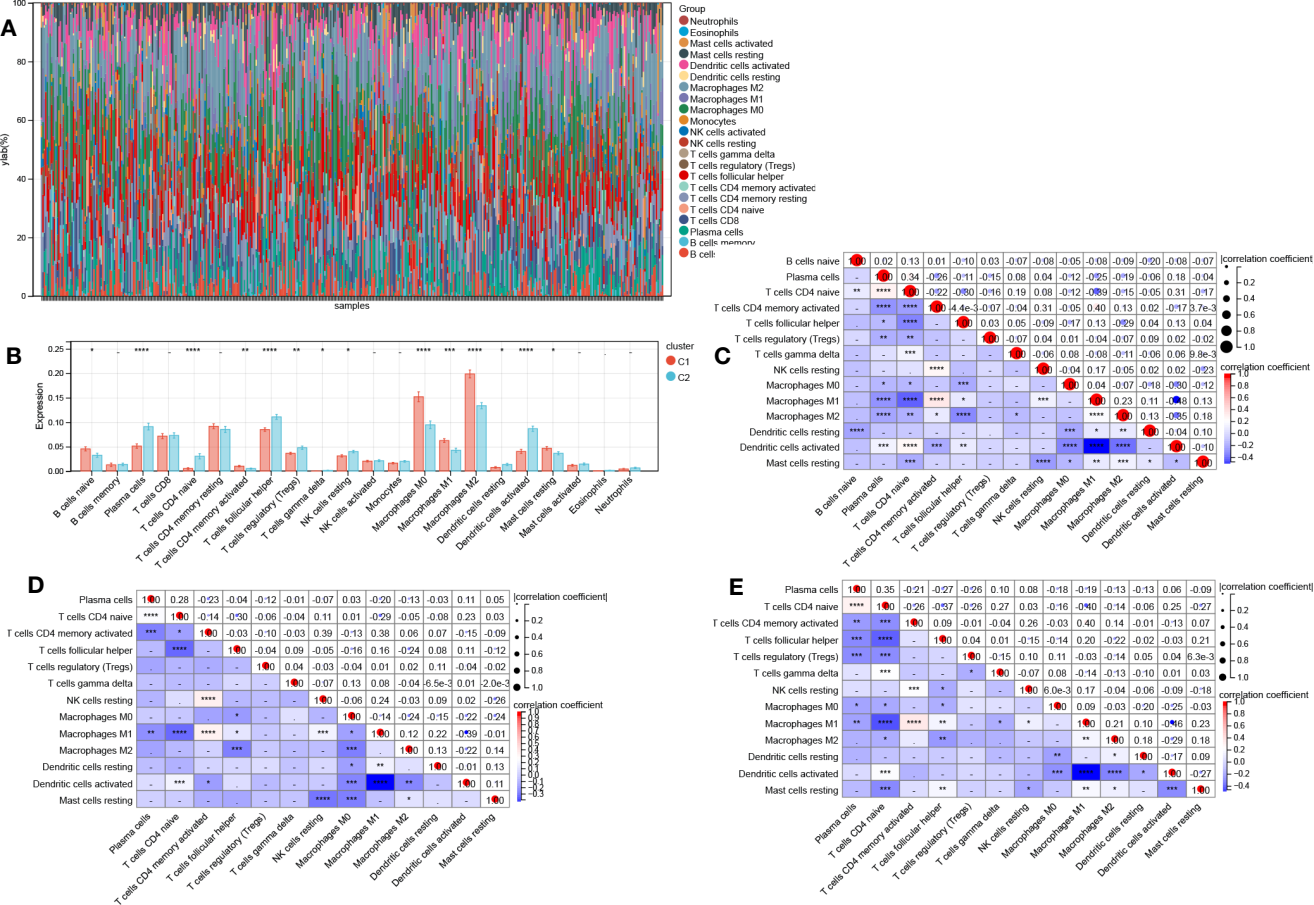
Differences in immune function across two aging subtypes. **(A)** Stacking plot of immune cell content for each sample **(B)** 22 immune cell types content histogram in two different aging patterns. Correlations between 14 immune cells in all patients **(C)**, clusters 1 patients **(D)**, and cluster 2 patients **(E)**. Data are expressed as means +/- SD. *P < 0.05; **P < 0.01; ***P < 0.001; ****P < 0.0001.

CIBERSORTx was used to estimate immune cell infiltration. **Figure 8A** shows the abundance of 22 distinct immune cells per sample. Total 14 immune cell contents differed between cluster 1 and cluster 2. The levels of mast cells resting, B cells native, M0, M1, and M2 macrophages, and T cells CD4 memory activated were substantially elevated in cluster 1 relative to those in cluster 2. Additionally, the levels of plasma cells, NK cells resting, T cells follicular helper, T cells gamma delta, T cells regulatory (Tregs), T cells CD4 native, dendritic cells (DCs) resting, and DCs activated were considerably lowered in cluster 1 in contrast with cluster 2 (**Figure 8B**).

**Figure 9:**
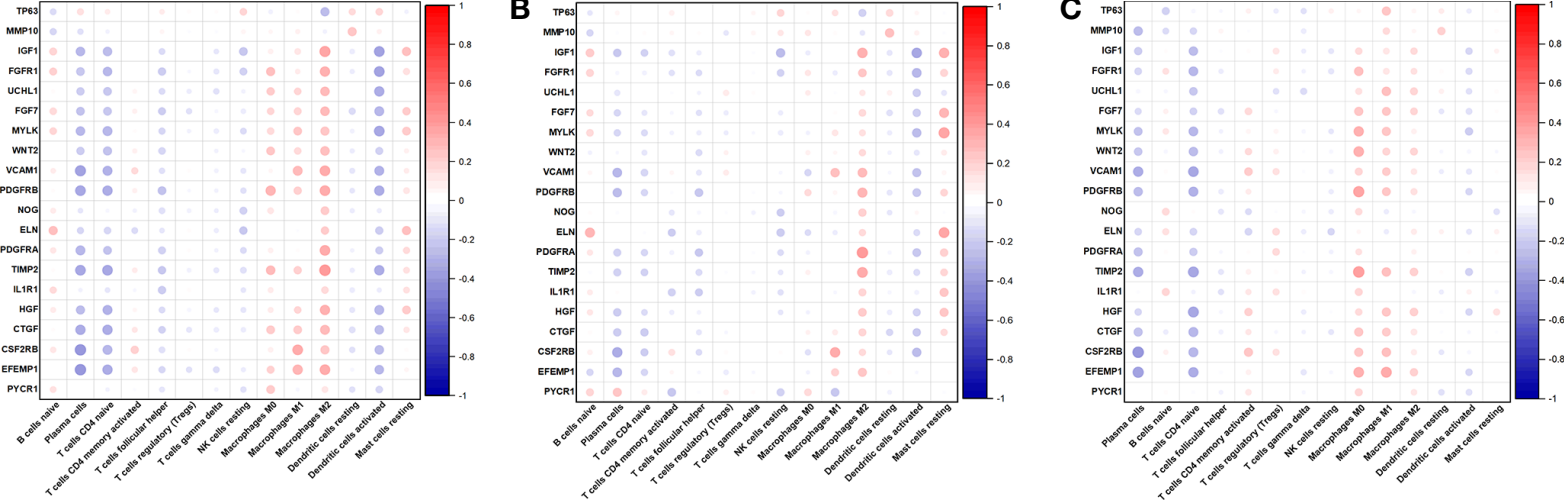
Correlations between 20 AR-DEGs and 14 immune cell types in all patients **(A)**, cluster 1 patients **(B)**, and cluster 2 patients **(C)**.

Then, we also determined the association between the 14 immune cell contents in all patients (**Figure 8C**), clusters 1 patients (**Figure 8D**), and cluster 2 patients (**Figure 8E**) was also calculated. Nonetheless, these correlations were weak. Last, we calculated the correlations between 20 AR-DEGs and 14 immune cell types in all patients (**Figure 9A**), cluster 1 patients (**Figure 9B**), and cluster 2 patients (**Figure 9C**). Plasma cells, T cells CD4 native, and DCs activated had negative correlations with AR-DEGs. M0, M1, and M2 Macrophages, as well as mast cells resting, had positive correlations with AR-DEGs.

### Expression level of TP63, ELN, EFEMP1, and UCHL1, and survival analysis

**Figure 10:**
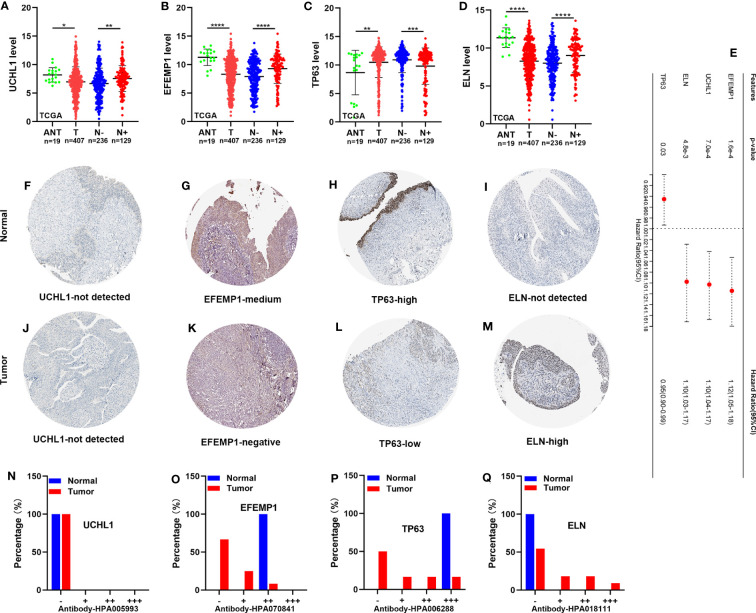
The expression level of TP63, ELN, EFEMP1, and UCHL1, and survival analysis. **(A-D)** The RNA expression of *UCHL1*, *EFEMP1*, *TP63*, and *ELN* in the TCGA cohort. **(E)** OS of BCa patients determined via univariate Cox regression analysis in TCGA. **(F-M)** IHC staining of UCHL1, EFEMP1, TP63, and ELN in normal tissues (up) and BCa tissues (down) in HPA. **(N-Q)** The proportion of BCa tissues with negative (−), weak (+), moderate (++), and strong (+++) staining intensity of UCHL1, EFEMP1, TP63, and ELN. HPA, the human protein atlas. *P < 0.05; **P < 0.01; ***P < 0.001; ****P < 0.0001.

For validation of TP63, ELN, EFEMP1, and UCHL1 expression in adjacent normal tissue (ANT), tumor tissues (T), lymphatic metastasis-negative tumor tissues (N-), and lymphatic metastasis-positive tumor tissues (N+), we acquired RNA and immunohistochemistry (IHC) findings from TCGA and the human protein atlas (HPA). *ELN, EFEMP1*, and *UCHL1* RNA expression levels were all elevated, whereas *TP63* RNA expression levels were decreased in N+ tissues relative to N- tissues (**Figures 10A–D**). This matches the result of their survival analysis (**Figure 10E**). Conversely, *ELN*, *EFEMP1*, and *UCHL1* were expressed at low levels, whereas TP63 was expressed at high levels in tumor tissues in contrast with ANT. The IHC staining analysis illustrated an elevated level of ELN in tumor tissues. However, EFEMP1 and TP63 were expressed at low levels in tumor tissues and at high levels in normal tissues. No significant variation was discovered in UCHL1 expression across various tissues (**Figures 10F–Q**).

### ELN recombinant protein facilitates BCa cell migration and invasion *in vitro*

**Figure 11:**
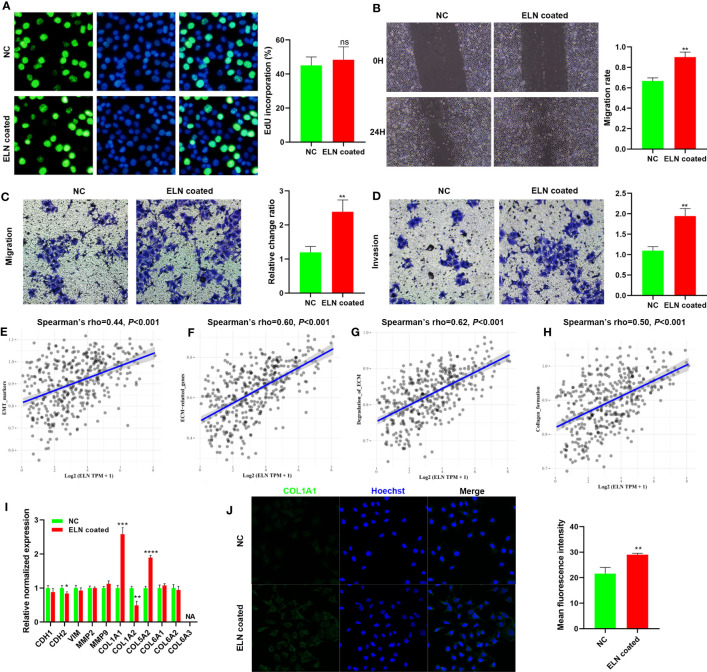
ELN recombinant protein facilitates BCa cell migration and invasion *in vitro*. Edu assays **(A)**, wound healing assays **(B)**, and Transwell assays **(C, D)** were conducted to determine the role of ELN. Correlations between ELN expression and EMT **(E)**, ECM **(F)**, degradation of ECM **(G)**, and collagen formation **(H)**. **(I)** qPCR was conducted to evaluate the expression of 11 specific target genes. **(J)** COL1A1 level was measured by immunofluorescence staining. Data are expressed as means +/- SD. *P < 0.05; **P < 0.01; ***P < 0.001; ****P < 0.0001.

SW780 cells were either treated with recombinant ELN protein or left untreated on growth plates for 48hrs to examine ELN’s role in BCa cells *in vitro*. First, to evaluate ELN’s function, we conducted Edu, wound healing, and Transwell assays. ELN recombinant protein significantly facilitated migration and invasion of BCa cells (**Figures 11A–D**). Then, we performed GSVA to explore the correlations between ELN expression and potential pathways, such as EMT, ECM, degradation of ECM, and collagen formation. As predicted, ELN was closely related to these pathways (**Figures 11E–H**). We selected 11 genes closely related to these pathways for validation. The results demonstrated that recombinant ELN protein promoted *COL1A1* and *COL5A2* expressions, and suppressed *CDH2* and *COL1A2* expressions (**Figure 11I**). **Figure 11J** shows that recombinant ELN protein promoted the protein level of COL1A1 in BCa cells (**Figure 11J**).

## Discussion

Our research is the first to discovered an ARGs signature from lymphatic metastasis BCa samples. We successfully constructed a four-ARG risk signature and identified two aging molecular subtypes with significant prognostic differences. At last, we investigated the role of ELN in BCa progression.

In the first step of our research, we identified 458 DEGs between lymphatic metastasis negative and positive samples. The annotation and enrichment analysis illustrated the primary enrichment of DEGs in ECM organization and organismal development. ECM consists of numerous structural proteins and is a dynamic network scaffold. Its function was determined by its composition, structure, and numerous post-translational modifications. Tumor invasion and metastasis is a complex multi-step biological process, specific to lymphatic metastasis that involves lymphangiogenesis, invasion of lymphatic vessels by malignant cells, a lymphatic system transfer of cancerous cells to lymph nodes, and settlement and lymph node colonization by tumor cells (20, 21). Each stage seems to be linked to the dynamic modulation of ECM. First, the ECM alterations may directly trigger increased cell malignancy (22, 23). Rose et al. (24) found that tumor suppressor ITIH5 affects cell adhesion, motility, and chemotherapeutic response by regulating the ECM, resulting in reduced BCa cell migration capacities. Proinflammatory cytokine OSM promotes breast tumor progression by modulating collagen fiber reorganization and alignment in stromal ECM (25). Alonso et al. (26) revealed that increases in ECM stiffness enhance tumor cell proliferation and invasion, and tumor-associated macrophages (TAM) and ECM stiffness work together to lead to an invasive phenotype and regulate the expression levels of critical indicators linked to EMT. Second, ECM remodeling around lymphatic vessels and lymph nodes can disrupt secondary lymphoid organs and their conduits. These changes, while disrupting the immune response, also create conditions for tumor cells to invade the lymphatic system.

Then, we identified 20 aging-related DEGs. The ECM organization and aging signaling pathways were particularly enriched for these DEGs. Initially, aging was thought to be analogous to apoptosis and autophagy, which can fight tumorigenesis by removing dysfunctional or diseased cells (27, 28). This view persisted until the discovery of senescence-associated secretory phenotype (SASP), the most important environmental effect of aging. SASP works by altering the TME, and the phenotypic features of microenvironmental senescence can be summarized as altered chemical signaling (29), disruption of ECM integrity (30–35), and immune senescence (36, 37). Thus, Whether ARGs can promote lymphatic metastasis by regulating ECM deserves further study. Finally, we successfully constructed a four-ARG risk signature. Patients’ prognoses can be effectively predicted using this risk signature.

We also presented a novel aging molecular subtype of BCa. Based on four signature ARGs, two aging molecular subtypes (cluster 1 and cluster 2) were discovered, and cluster 1 possessed an unfavorable OS in contrast with cluster 2. Traditional histological classification has certain limitations, especially for high-grade tumors, chemotherapy-resistant tumors, and recurrent tumors. We can more accurately predict the prognosis of bladder cancer by combining molecular classification with the histopathological diagnosis (38, 39). We found that classical tumor signaling pathways, ECM-related signaling pathways, and immune-associated signaling pathways were differentially expressed in the molecular BCa subtypes. This provides a new perspective on the occurrence and progression of BCa. Moreover, the risk score and four signature ARGs had good classification efficacy for two aging molecular subtypes. This proves that our risk signature has good clinical value.

We assessed immune cell infiltration using GSEA data obtained by comparing two aging subtypes. A total of 14 immune cell, including 5 kinds of T cells, contents differed between the two subtypes in all patients. This may be a reason of dismal prognosis among C1 patients. T cells have major impact on patient outcomes (40). David Y Oh et al. (41) reported that CD4+ T cell can kill autologous tumors in BCa. The high CD8+ T cell infiltration was significantly associated with longer cancer-specific survival of the patients with BCa (42). Lymphangiogenesis is also regulated by immune cells. Tumor-associated macrophages (TAMs) serve as prominent metastasis promoters in tumor progression (43). Huang et al. (44) demonstrated that the HSF1-PRMT5-WDR5 axis promoted macrophages infiltration to facilitate multistep lymphatic metastasis in BCa. In this study, we found macrophages were remarkably increased in cluster 1 relative to cluster 2. In addition, researchers found that neutrophil infiltration was increased in lymphatic metastasis BCa and associated with poor prognosis (45). However, our results showed that neutrophils infiltration did not differ among molecular subtypes.

*ELN*, *EFEMP1*, and *UCHL1* were expressed at low levels whereas TP63 was expressed at high levels in tumor tissues in contrast with ANT. This does not match the result of their survival analysis suggesting that assessing its expression in tumor tissue is insufficient to determine its role in cancer. The enzyme ubiquitin C-terminal hydrolase L1 (UCHL1), which is part of the deubiquitinase (DUB) family of enzymes, acts as an oncogenic promoter or a tumor suppressor depending on the specific type of cancer being considered (46–49). Liu et al. (50) revealed that UCHL1 regulated the cell cycle and EMT to promote BCa cell proliferation, migration, and invasiveness. Tumor Protein 63 (TP63) encodes a member of the p53 family of transcription factors and can function both as a tumor suppressor and an oncogene (51–53). Bankhead et al. (54) found that the complete opposite role of TP63 was due to its differential isoform expression. Expression of the TAp63 isoform was correlated with a better prognosis. However, the expression of the DNp63 isoform was linked to the luminal subtype and had a dismal prognosis. EGF Containing Fibulin Extracellular Matrix Protein 1 (EFEMP1) and Elastin (ELN) encode proteins that make up the ECM. There is research evidence linking them to tumor invasiveness and metastasis. Ying et al. (55) proved that the progression of BCa is promoted by the METTL1-m (7)G-EGFR/EFEMP1 axis. Chen et al. (56) confirmed that elevated levels of EFEMP1 immunoexpression were shown to be substantially linked to advanced BCa pathological stage, higher histological grade, lymph node metastases, perineural invasion, excessive mitosis, and vascular invasion. Additionally, EFEMP1 dysregulation substantially enhanced the actin cytoskeleton signaling pathway, the TME pathway, and mitochondrial dysfunction. Salesse et al. (57) revealed that the severity of illness and patient age are both predictive with a higher ELN and the invasive potential of breast cancer cells may be aided by elastosis and/or an aged stroma. Li et al. (58) also found that ELN is an essential component in the progression of colorectal cancer. The expression level of the ELN gene was found to be elevated in colorectal cancer patients, suggesting that ELN could have triggered EMT.

Because ELN was one of the most common ECM components, we further evaluated its role through a series of functional experiments in BCa cells. We found that ELN recombinant protein facilitates BCa cell migration and invasion *in vitro*. GSVA results revealed that ELN was closely related to EMT, ECM, degradation of ECM, and collagen formation. Therefore, we selected EMT markers (*CDH1*, *CDH2*, *VIM*), collagen-related genes linked to BCa prognosis (57) (*COL1A1*, *COL1A2*, *COL5A2*, *COL6A1*, *COL6A2*, and *COL6A3*), and ECM degradation related genes (*MMP2*, *MMP9*) for verification. Our results demonstrated that recombinant ELN protein promoted *COL1A1* and *COL5A2* expressions, suppressed *CDH2* and *COL1A2* expression, and promoted the protein level of COL1A1. Zhu et al. (59) confirmed that COL1A1 knockdown significantly attenuated the proliferative, migratory, and invasive capacities of 5637 and T24 cells. This effect was achieved by inhibiting the EMT process and the TGF-β signaling pathway. Their mechanism of action requires further in-depth study in lymphatic metastasis in BCa.

In summary, we established a novel four-ARG risk signature based on lymphatic metastasis samples, and this signature could serve as an effective survival predictor for patients with BCa.

## Data availability statement

The original contributions presented in the study are included in the article/**Supplementary Material**. Further inquiries can be directed to the corresponding author.

## Author contributions

(I) Conception and design: ZZ, ZL, XL. (II) Administrative support: ZZ, ZL. (III) Provision of study materials or patients: ZL, XL. (IV) Collection and assembly of data: DL. (V) Data analysis and interpretation: ZZ. (VI) Manuscript writing: ZZ, XL. All authors contributed to the article and approved the submitted version.
