# Supplementary material for: A novel signature of aging-related genes associated with lymphatic metastasis for survival prediction in patients with bladder cancer
Source: Front Oncol. 2023 Jun 27;13:1140891. doi: 10.3389/fonc.2023.1140891 (PMC10335803; doi:10.3389/fonc.2023.1140891)
Supplement: Supplementary file 4 [file Table_1.docx]

| Genes | Forward primer (5'->3') | Reverse primer (5'->3') |
| --- | --- | --- |
| MMP2 | GATACCCCTTTGACGGTAAGGA | CCTTCTCCCAAGGTCCATAGC |
| MMP9 | TGTACCGCTATGGTTACACTCG | GGCAGGGACAGTTGCTTCT |
| CDH1 | CGAGAGCTACACGTTCACGG | GGGTGTCGAGGGAAAAATAGG |
| CDH2 | AGCCAACCTTAACTGAGGAGT | GGCAAGTTGATTGGAGGGATG |
| VIM | AGTCCACTGAGTACCGGAGAC | CATTTCACGCATCTGGCGTTC |
| COL1A1 | GAGGGCCAAGACGAAGACATC | CAGATCACGTCATCGCACAAC |
| COL1A2 | GGCCCTCAAGGTTTCCAAGG | CACCCTGTGGTCCAACAACTC |
| COL5A2 | GACTGTGCCGACCCTGTAAC | CCTGGACGACCACGTATGC |
| COL6A1 | ACAGTGACGAGGTGGAGATCA | GATAGCGCAGTCGGTGTAGG |
| COL6A2 | TACGGAGAGTGCTACAAGGTG | GGTCCTGGGAATCCAATGGG |
| COL6A3 | CATAACCGCTGTGCGGAAAAT | TCATCTAGGGACTTACCACCTG |
| GADPH | ACAACTTTGGTATCGTGGAAGG | GCCATCACGCCACAGTTTC |

Table S1 Primer sequence
